# Granuloma annulare with alopecia areata in a 6-year-old girl: a case report

**DOI:** 10.1186/s13256-023-03864-7

**Published:** 2023-05-11

**Authors:** Fatemeh Mohaghegh, Reza Moeine, Mina Saber, Safdarian Fatemeh, Mohammad Nekooeian, Reza Shahriarirad

**Affiliations:** 1 Department of Dermatology, Skin Diseases and Leishmaniasis Research Center, Isfahan University of Medical Sciences, Isfahan, Iran; 2 Clinical Research Development Center of Children Hospital, Hormozgan University of Medical Sciences, Bandar Abbas, Iran; 3 Health and System Research Center, Shiraz University of Medical Sciences, Shiraz, Iran; 4 Thoracic and Vascular Surgery Research Center, Shiraz University of Medical Sciences, Shiraz, Iran; 5 School of Medicine, Shiraz University of Medical Sciences, Shiraz, Iran

**Keywords:** Granuloma annulare, Alopecia areata, Pediatric, Dermatology

## Abstract

**Background:**

Dermatologic signs and symptoms can be the manifestations of a single disease or different diseases, and it is proven that some are associated with one another. These connections are not fully understood, but the answer lies in the pathophysiology of each disease.

**Case presentation:**

We report the case of a 6-year-old Middle-Eastern girl who presented with two skin lesions on the dorsum of her foot, along with scaling of her soles and palms, face skin discoloration, and areas of patchy alopecia on her scalp. She was diagnosed as a case of acute onset of granuloma annulare with alopecia areata and dermatitis. The treatment regimen for the patient’s scalp consisted of topical minoxidil and betamethasone and three sessions with 1-month intervals of triamcinolone acetonide intralesional injections, which demonstrated modest effectiveness in treating alopecia areata.

**Conclusion:**

Granuloma annulare is a benign inflammatory illness with no known cause that might be difficult to cure. The clinical course and prognosis might vary greatly depending on the disease subtype, and associating symptoms and diseases, such as alopecia areata, should be considered.

## Introduction

Granuloma annulare (GA) is a benign cutaneous idiopathic condition, consisting of generalized and localized forms with polymorphic manifestations [1]. GA has been linked to thyroid disease, diabetes, collagen vascular disease, dyslipidemia, infectious hepatitis, malignancies, and systemic infections [2]. However, the concurrence of GA with other dermatological conditions has rarely been reported.

Although the exact etiology of alopecia areata (AA) has not been fully explained, many reports suggest that it is an organ-specific autoimmune disorder targeted at hair follicles. Nevertheless, the mechanisms, antigenic targets, and consequences of the autoimmune attack in AA are not yet fully understood [3, 4].

The relationship between GA and AA has been reported coincidentally in previous reports [5]. Some dermatologic conditions are primary skin diseases. Others turn out to be a symptom or a manifestation of secondary disease, and about 10% of patients can have more than one skin disease simultaneously [6, 7]. Furthermore, the presence of chronic medical illness and use of steroids may worsen the prognosis of immunological disease [8, 9]. However, herein we report a case of a 6-year-old girl with an accompanying presentation of GA and two autoimmune conditions: AA and an unusual presentation of dermatitis.

## Case presentation

**Fig. 1:**
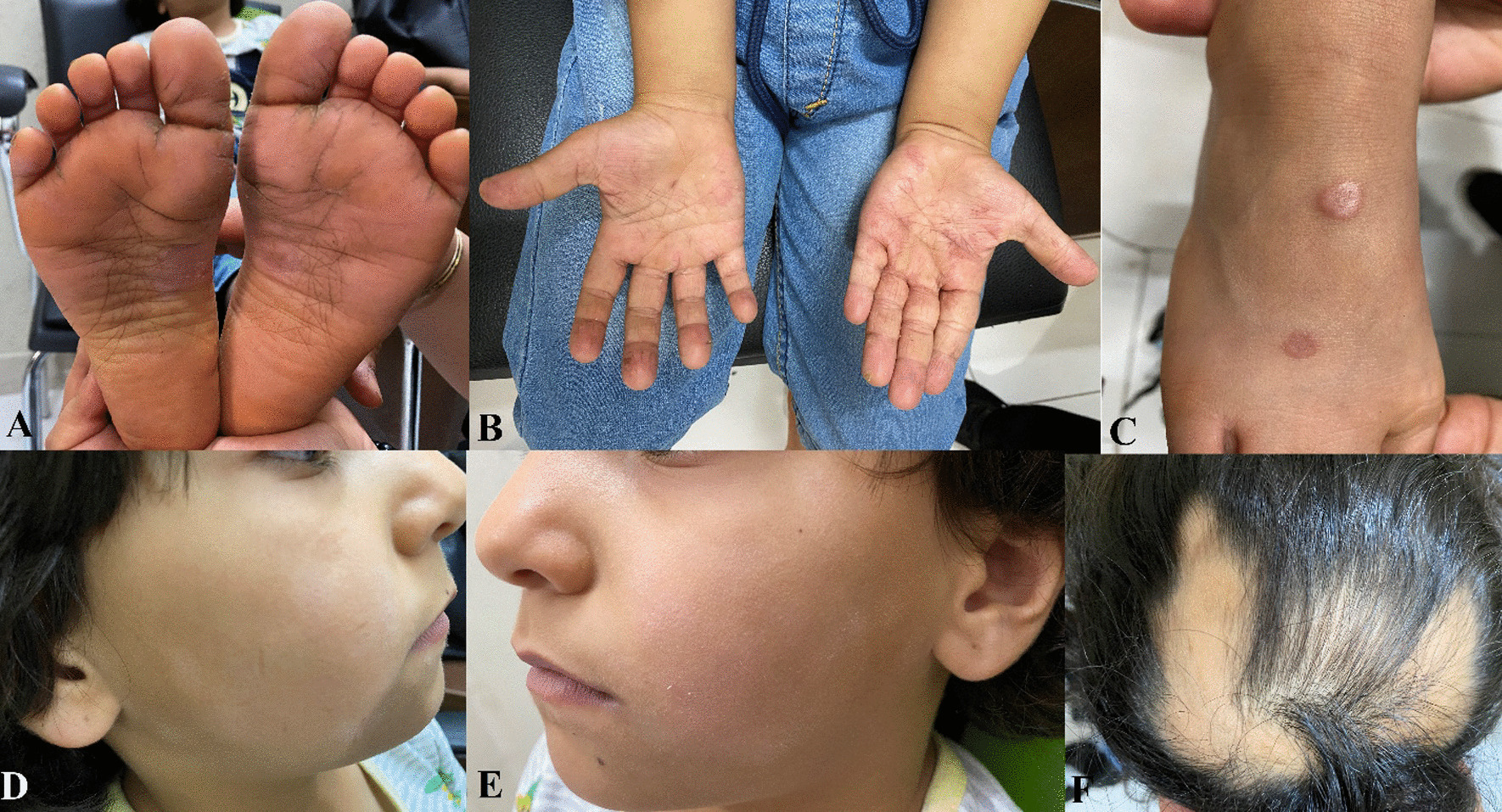
A 6-year-old girl with (**A**) eczema, scaling, and hyper linearity of the foot; (**B**) multiple erythematous, pruritic rashes over both palms; (**C**) red-to-pink colored nodules on the dorsal aspect of the right foot in favor of granuloma annular; (**D** and **E**) hypopigmented patches over the buccal area with fine scaling suggestive of pityriasis alba; (**F**) patchy alopecia with well-defined borders

A 6-year-old Middle-Eastern girl first came to a dermatology clinic with two skin lesions on the dorsum of her foot, along with scaling of her soles and palms, face skin discoloration, and areas of patchy alopecia on her scalp (Fig. 1). The patient’s past medical and family history was insignificant for dermatological disease. As claimed by the patient’s mother, the patient first noticed the presence of some eczematous-like lesions on her soles. These lesions were initially limited to soles; however, they spread to her palms and the ventral aspect of her fingers in a few days. Shortly after, she presented with face skin hypopigmentation, followed by the emergence of some alopecic areas on her scalp. Eventually, two lesions with distinct borders grew on the dorsal aspect of her foot.

**Fig. 2:**
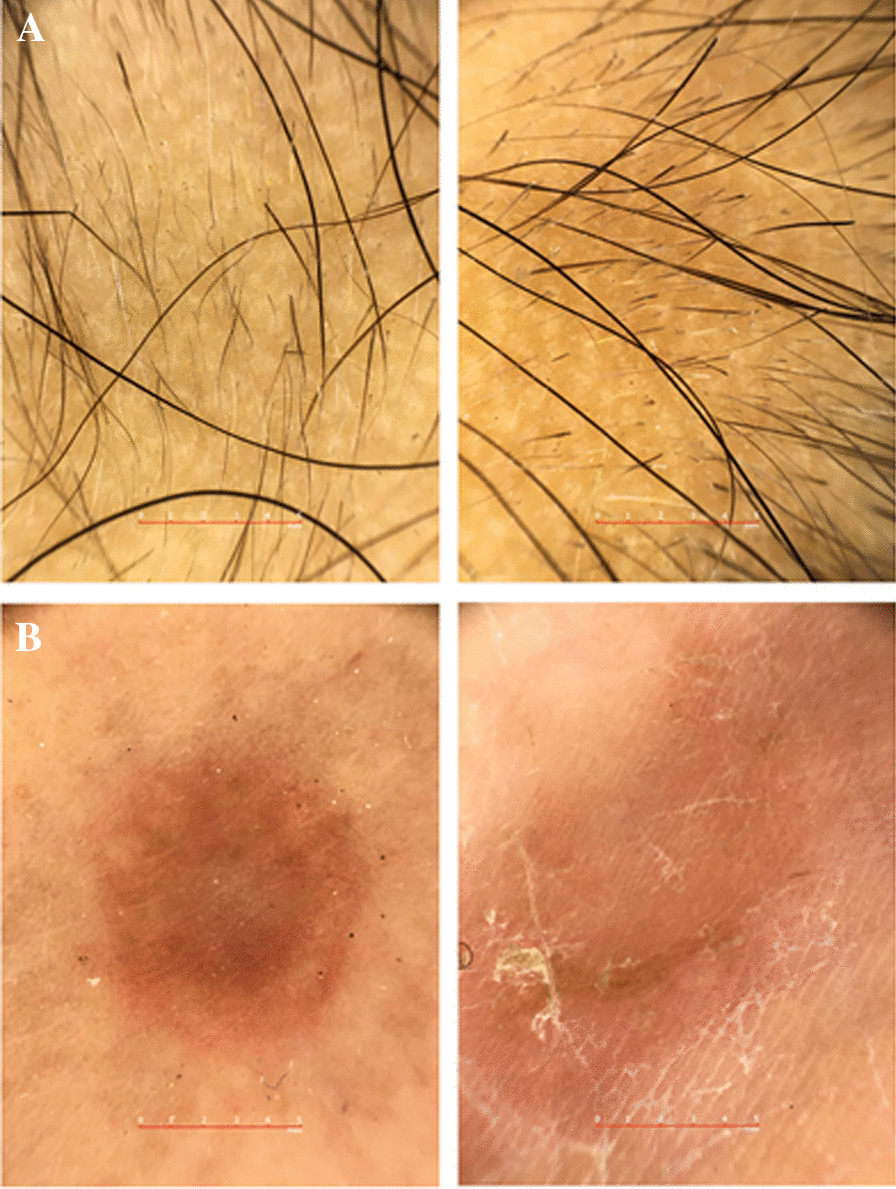
Dermoscopy evaluation of granuloma annulare, alopecia areata, and palm eczematous lesions of a 6-year-old girl demonstrating unfocused vessels, pinkish–reddish background, and yellowish-orange structureless areas

On physical examination, there were areas of patchy alopecia with well-defined borders over the scalp, mainly on the parietal part, accompanied by a characteristic finding of alopecia areata, exclamation marks in trichoscopy evaluation. In the areas of alopecia, there was no scalp erythema, inflammation, or scaling. The rest of the physical examination showed multiple erythematous, pruritic rashes over both palms, soles, and ventral aspect of fingers. In some areas, these lesions became painful and ulcerated with white scaling. Furthermore, hyperlinearity was found in both soles and palms. In addition to these findings, two distinct red-to-pink colored nodules on the right foot’s dorsal aspect, which favored granuloma annular, were examined. On the face, physical examination revealed symmetrical hypopigmented patches mostly over the buccal area with fine scaling, suggestive of pityriasis alba. Dermoscopic evaluation of the patient’s granuloma annulare lesions demonstrated unfocused vessels, pinkish–reddish background, and yellowish-orange structureless regions (Fig. 2). Dermoscopic findings of the scalp in the areas of alopecia revealed some characteristic features of alopecia areata, including black and yellow dots and multiple exclamation marks. Palms and soles dermoscopy exhibited pink background accompanied by scattered dot vessels and yellow or white scaling.

Eventually, the patient was diagnosed with granuloma annular, alopecia areata, and pityriasis alba. The patient was first prescribed topical mometasone twice a day for 1 week, emollients, and mild cleansing soaps to treat eczematous palms and soles lesions, and was maintained with calcineurin inhibitors. Concerning nodules on the foot dorsum, mometasone cream twice daily was started. Moreover, the treatment regimen for the patient’s scalp consisted of topical minoxidil and betamethasone and three sessions with 1-month intervals of triamcinolone acetonide intralesional injections, which demonstrated modest effectiveness in treating alopecia areata.

**Fig. 3:**
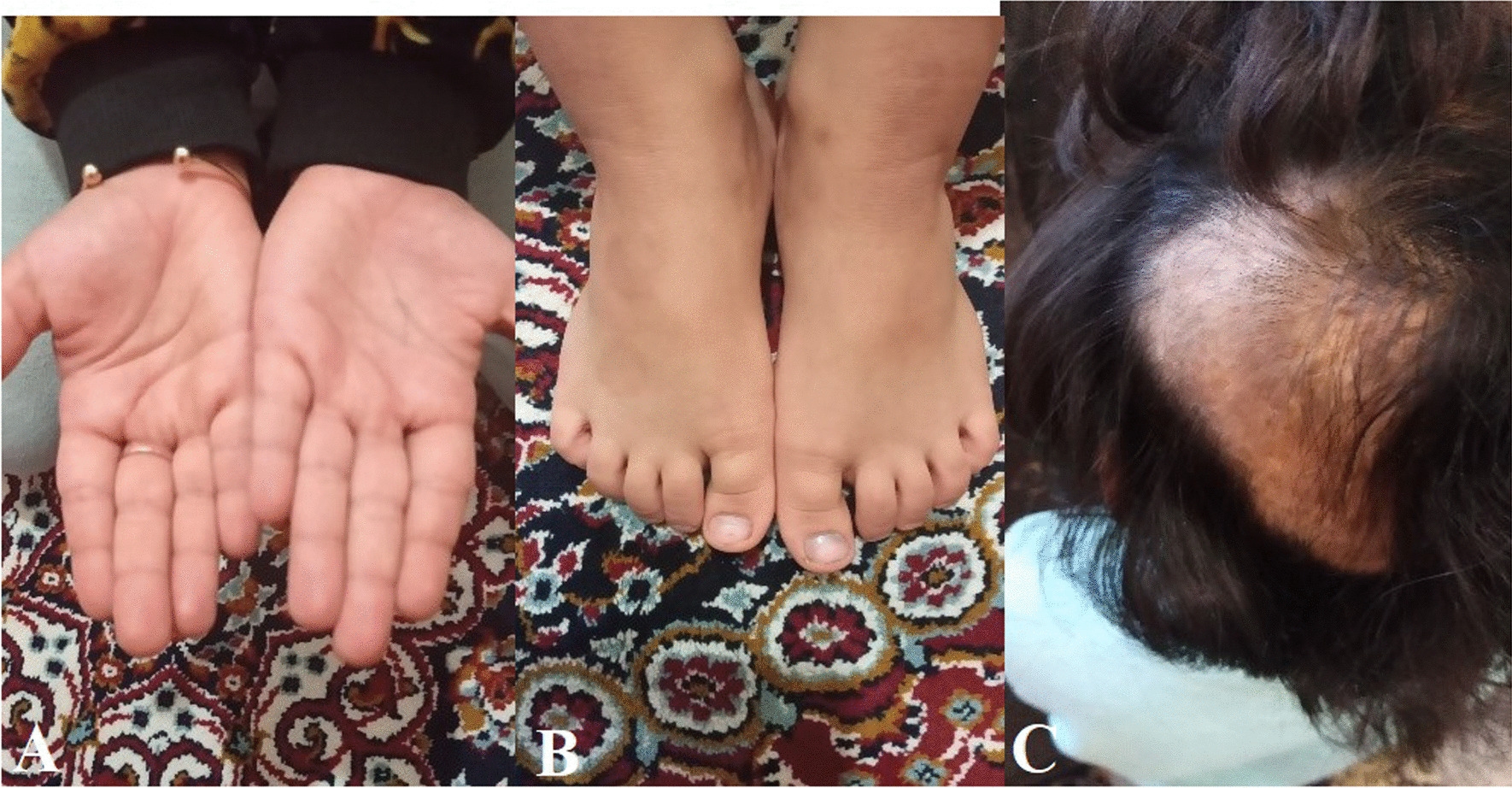
Patient’s palms lesions (**A**), granuloma annulare lesions (**B**), and alopecia areata (**C**) after 3 months of pharmacotherapy

The patient’s palms and feet responded well to pharmacotherapy during follow-up (Fig. 3); therefore, the topical steroid prescribed for palms and soles lesions had been gradually tapered, and the patient was symptom-free with topical emollients. Granuloma annulare lesions responded well to treatment. However, areas of alopecia were still present on the patient’s scalp.

## Discussion

Dermatologic conditions account for about a quarter of visits to pediatric clinics each year [10, 11]. These pathologies can be concomitant or have two or more different etiologies. Herein, we reported a rare case of four different conditions that manifested shortly one after another. Our case’s first signs of dermatologic condition were erythema and scaling and hyperlinearity of palms and soles followed by patchy buccal hypopigmentation, which was in favor of dermatitis and pityriasis alba. Pityriasis alba is usually considered a minor manifestation of atopic dermatitis and is rarely seen as a different entity [12].

Atopic dermatitis is a relatively common skin condition in children. There are four primary criteria for diagnosis of atopic dermatitis: pruritic nature, characteristic involvement of the head and flexor areas in younger children and extensor areas in older children and adults, chronic and relapsing coarse, and personal and familial history of atopy [13]. On the basis of the patient’s physical examination, the findings did not meet the criteria of atopic dermatitis. They did not have a history of other allergic conditions such as asthma, known as the atopic march. Regarding the involvement pattern of eczema in our case, a study by Agner *et al*. concluded that concomitant eczema of hands and feet is not uncommon and can be seen in different pathologies. Aside from atopic dermatitis, these other differentials include hyperkeratotic hand eczema, irritant or contact allergic dermatitis, and hyperkeratotic endogenous eczema [14, 15]. In addition, atopic dermatitis should be considered in children with a relapsing or chronic pruritic dermatitis, distributed on the face and extensor surfaces in young children and infants, or involving flexural surfaces in adolescents and older children [16].

Shortly after eczema, the patient developed AA, considered an autoimmune disease. Up to 20% of the cases occur in children, and the peak incidence of this disease is 2–6 years old in pediatrics [17]. Previous studies have shown that dermatitis, thyroid disease, and other autoimmune diseases such as vitiligo are associated with an increased risk of AA [18, 19]. Furthermore, AA has also been seen as a rare side effect following TNF-α inhibitor therapy with adalimumab [20].

The last condition seen in this patient was the well-defined nodules on the foot’s dorsal aspect. The appearance and location of the lesions were in concordance with the diagnosis of localized GA, which was confirmed with dermoscopy. These benign lesions can be seen in children and adults and are self-limited [21, 22]. This condition is associated with diabetes mellitus, autoimmune conditions such as thyroid disease, and hyperlipidemia, among other pathologies [23]. The proposed pathology is a delayed-type hypersensitivity reaction, but the connection of GA to the other conditions is not fully clear. In a similar report of a 4-year-old girl, AA developed a few months after the appearance of GA plaques on hands and feet [24]. In addition, AA and granuloma annulare were seen together in a case of Malassezia (Pityrosporum) folliculitis in a 35-year-old man, which was considered coincidental [5]; however, data regarding similar cases are lacking.

Currently, there is a lack of literature and evidence regarding concurrence of these diseases and most reports mentioned this overlapping as coincidental. Whether or not these is a physiopathological link between these entities is still a matter of debate and requires further investigation; however, we believe that case reports such as ours could help shed light on this possibility and encourage further investigations, along with increasing diagnostic for dermatologists worldwide.

## Conclusion

Granuloma annulare is a benign inflammatory illness with no known cause that might be difficult to cure. The clinical course and prognosis might vary greatly depending on the disease subtype, and associating symptoms and diseases, such as alopecia areata, should be considered.

## Data Availability

All data regarding this study has been reported in the manuscript. Please contact the corresponding author if you are interested in any further information.
